# Long term neurological sequela of isolated infarctions according to the topographic areas of thalamus

**DOI:** 10.3906/sag-2006-146

**Published:** 2021-08-30

**Authors:** Aygül TANTİK PAK, Zahide MAİL GÜRKAN, Sebahat NACAR DOĞAN, Yıldızhan ŞENGÜL

**Affiliations:** 1 Department of Neurology, Gaziosmanpaşa Training and Research Hospital, İstanbul Turkey; 2 Department of Radiology, Gaziosmanpaşa Training and Research Hospital, İstanbul Turkey

**Keywords:** Thalamus infarction, cognitive impairment, anterior nucleus, variative areas, classical areas

## Abstract

**Background/aim:**

Thalamus infarctions presented with various clinical findings are considered to be related to classical and variative infarction areas. In our study, we aimed to compare the sequela clinical findings of patients with isolated thalamus infarction according to anatomical areas.

**Materials and methods:**

Seventy patients diagnosed with isolated thalamus infarction in our clinic between 2010 and 2020 were included in the study. The infarction areas of the patients were divided into groups by the radiologist, including the variative areas to the classical areas using magnetic resonance imaging. Neurological examinations were performed and recorded. Sequela clinical findings of the groups were compared.

**Results:**

The mean age of all patients was 64.49 ± 13.75 (range between: 33–81) years, and the female ratio was 52.9% (n: 33). Inferolateral area infarction was detected most commonly. The most common complaints were sensory complaints (48.6%), speech disorders (20%), limb weakness (15.7%). There were no significant association between the neurological examination findings of classical and variative area infarctions of patients whose most common admission complaint is sensory deficits (p < 0.05), and significant signs of cognitive impairment were detected in the anterior area compared to other areas (p < 0.001). It can be considered that cognitive impairment we detected in the anterior area developed due to its associations.

**Conclusion:**

In our study where sequela findings were evaluated, the absence of a significant difference in neurological examination findings can be explained by the decline of many acute clinical findings over time.

## 1. Introduction

Thalamus plays a role in many high-level neurological functions, including the transfer of sensory and motor signals to the cerebral cortex, regulation of consciousness, sleep and wakefulness. Classical areas of thalamus are anterior, paramedian, inferolateral and posterior areas [1,2]. However, with the development of imaging methods, it has been reported that there are also variational areas. These areas are 1) Anteromedian region: defined as the infarctions including the posterior of the anterior region and anterior of the paramedian region. 2) Central region: The region which is defined with the inclusion of parts of four adjacent regions in the middle part of the thalamus. 3) Posterolateral region: It is defined as the area connecting the posterior part of the inferolateral region and the anterior of the posterior region. Thalamus is fed by four arteries (polar, thalamoperforating, thalamogeniculate, and posterior choroidal arteries) and the topographic regions of the thalamic infarctions are classically classified according to the watershed area of these four arteries. Variative areas are considered to develop as a result of anatomical variation of these arteries or border zone infarcts [3,4]. 

Thalamus contains many nuclei, and these nuclei are affected at various rates according to vascular lesions. Patients who admitted to the hospital with thalamus infarction may apply with hemiparesis, hemihypoesthesia, visual field defect, consciousness disorders, sleep disorders, neuropsychiatric findings. Information about the topographic area lesions of the thalamus and their clinical correlations develop after postthalamotomy or with infarction area-clinical finding correlations [5]. Because of the complex nucleus structure and arterial variations, information about thalamus needs to be increased in order to be able to identify patients with different clinical findings, to detect etiological causes and to treat them appropriately.

Our hypothesis is that the reason of clinic finding of patients presenting with thalamus infarction are different from each other is related to the variation of arteries or variative infarction areas developing with border zone infarcts. In the literature, there are studies investigating the etiological causes and neurological findings of classical and variative areas, however there are no studies evaluating long-term sequela findings according to their anatomical regions. In this study, we aimed to detect the sequela findings of isolated thalamus infarctions according to the anatomical areas of the thalamus (classical and variative areas). 

## 2. Materials and methods

Our study was planned as a retrospective study. The study was conducted in accordance with the ethical principles stated in the “Helsinki Declaration” and approved by Gaziosmanpaşa Training and Research Hospital Ethics Committee. Informed written consent was obtained from the participants after the characteristics of the procedures were fully explained.

### 2. 1. Patients 

This study was conducted between January and March 2020, and 140 patients who were hospitalized, investigated and treated in our hospital’s neurology inpatient clinic between 2010 and 2020 and diagnosed with isolated unilateral thalamus infarction with magnetic resonance imaging (MRI) were included. Our inclusion criterion was to have unilateral isolated thalamus infarction. Among these patients, 23 were excluded because of exitus, 26 were excluded because we could not reach them and 21 patients were excluded because they had recurrent ischemic stroke or hemorrhagic stroke. As a result, the study was conducted with 70 patients. Our exclusion criteria were to have bilateral thalamus infarction, recurrent ischemic stroke, hemorrhagic stroke, neurodegenerative comorbid disease. Total of seventy patients completed the study protocol entirely. Patients’ sociodemographic data, time of stroke,stroke etiologies according to TOAST classifications [6] and sequela neurological findings were recorded. Mini-mental state examination (MMSE) [7] of forty-two patients and the neurological examinations of the all patients which were conducted in the last three months were noted based on follow-up notes. MRI examinations were evaluated by radiologist (SND) and infarction areas (classical and variative areas) were determined. Clinical findings and etiological causes were compared according to infarction areas.

### 2. 2.Mini-mental state examination (MMSE)

MMSE was first published by Folstein et al [7]. Turkish validity and reliability study was carried out by Güngen et al. in 2002 [8]. Test is a short, useful and standardized method that can be used to determine the cognitive level. It consists of eleven items grouped under five main headings: orientation, recording memory, attention and calculation, recall, and language, and is evaluated over a total score of 30. 

### 2. 3. Imaging protocols 

Diffusion-weighted imaging’s (DWI) were obtained using two 1.5 T-magnetic resonance imaging (MRI) units (GE Signa HDxt and Signa Explorer; GE, Milwaukee, WI, USA). DWIs were acquired in the axial plane with parameters field of view: 25 mm, repetition time: 5000 ms, echo time: 100 ms, acquisition time: 1, number of excitations: 1, and b values of 0 and 1000 s/mm2, isotropically weighted. DWI yielded 20 contiguous slices that were 7 mm thick and axial-oblique. Apparent diffusion coefficient (ADC) map was automatically generated from DWI at b = 0 and b = 1000 s/mm2. The ADC maps were checked to make sure “real” diffusion disturbance. A visual evaluation was performed.

### 2. 4. Imaging analysis 

All DWIs were evaluated by a radiologist with significant experience in neuroradiology (S.N.D. with 11 years in neuroradiology) on a PACS imaging workstation (Infinitt PACS; Infinitt Healthcare, Seoul, Republic of Korea). The radiologist was blinded to neurologic symptoms during the retrospective imaging review. All DWIs were reviewed with regard to location of thalamic infarction based on previously template of classical and variative thalamic territories.

### 2. 5. Topography of thalamic Infarctions

Classic thalamic infarctions were assigned four vascular zones based on previously published territory templates [5,9,10] anterior, paramedian, inferolateral, and dorsal. Variative thalamic infarctions were assigned into three vascular zone on previously published territory templates [3,11] anteromedian, central, and posterolateral. 

### 2. 6. Statistics 

All statistical analyses were performed using a commercially available SPSS release 20.0 software package (IBM Corp., New York, NY, USA). When evaluating the data, in addition to descriptive statistical methods, frequency, percentage, mean, and standard deviation were used to determine the demographic characteristics of the cases.  Categorical variables were compared with chi-square test and continuous variables were compared either Student’s t-test or Mann–Whitney U test according to distribution normality. ANOVA was used for comparison of age, disease time, MMSE according to infarction areas and posthoc test was used for intergroup evaluation of MMSE. Again, ANOVA test was used to compare the nuclei of the thalamus with the subgroups of the mini-mental test. Posthoc Bonferroni analysis was performed. A p value of less than 0.05 was considered significant.

## 3. Results

**Figure 1:**
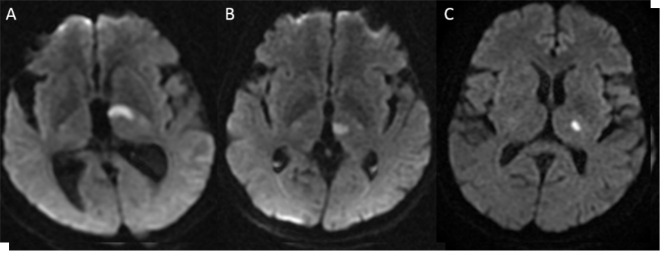
The classic territories of thalamus on DWI: anterior (A), paramedian (B), inferolateral (C). The isolated posterior territory infarction was not observed in present study.

**Figure 2:**
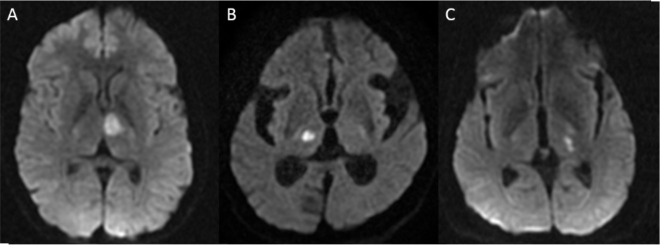
The variative territories of thalamus on DWI: anteromedian (A), central (B), and posterolateral (C).

The mean age of all patients was 64.49 ± 13.75 (ranged between: 33–81) years, the female ratio was 52.9% (n: 33) and the male ratio was 47.1% (n: 33). The mean time passed since the development of disease was 3.46 ± 2.35 years for all patients. While 72.9% (n: 51) of the patients had an infarction in the classical area of the thalamus (As shown in Figures 1A–1C), 27.1% (n: 19) had a variative area infarction (As shown in Figures 2A–2C). When the areas are evaluated topographically; infarction ratios were as follows; anterior area 20% (n: 14), paramedian area 5.7% (n: 4), inferolateral 50% (n: 35), anteromedian 4.3% (n: 3), central 8.6% (n: 6), posterolateral 8.6% (n: 6). Posterior area infarction was not observed in our patients. According to the ischemic stroke etiologies, 20% (n: 14) of the patients had major vascular disease, 17.14% (n: 12) had cardio embolic stroke, 10% (n: 7) had small vascular disease, 11.43% (n: 8) had other etiologies and 41.42% (n: 29) were in the group of undetermined cause (Table 1).

**Table 1:** Sociodemographic and clinical data of all patients.

	All patients (n: 70)
Age (years)	64.49 ± 13.75
SexFemaleMale	52.9% (n: 33)47.1% (n: 33)
Disease development time (years)	3.46 ± 2.35
Infarction areaClassicalAnteriorParamedianInferolateral Variative AnteromedianCentralPosterolateral	72.9% (n: 51)20% (n: 14)5.7% (n: 4)50% (n: 35)27.1% (n: 19)4.3% (n: 3)8.6% (n: 6)8.6% (n: 6)
Etiological causesMajor vascular diseaseCardio embolic strokeSmall vascular disease Other causesUndetermined	20% (n: 14)17.14% (n: 12)10% (n: 7)11.43% (n: 8)41.42% (n: 29)
Complaints (Most commonly reported %)HemihypoesthesiaSpeech disorderHemiparesisImpaired consciousness	48.62015.77.1

When the complaints of the patients when they first presented to the hospital with infarction were analyzed, the most common complaints were sensory complaint (48.6%), speech disorder (20%), limb weakness (15.7%), impaired consciousness (7.1%), dizziness (4.3%) and gaitimpairment (2.9%). When they admission complaints are analyzed according to infarction areas: the most common complaints in the admission were numbness (54.3%) in the inferolateral area; impaired consciousness (35.7%) in the anterior area; sensory complaint (hypoesthesia, numbness, tingling) (62.5%) in the central area (p = 0.029). Prominent additional complaints included a patient with inferolateral infarction experiencing decreased libido and another patient with anterior infarction complaining of difficulty swallowing. Cognitive evaluation performed with MMSE showed that anterior area was prominently affected than other areas (p < 0.001). However, there was no significant difference in the distribution of other neurological examination findings in the infarction areas (p > 0.05) (Table 2).

**Table 2:** Evaluation of sequela neurology findings according to vascular infarction areas.

	Anterior	Paramedian	Inferolateral	Anteromedian	Central	Posterolateral	p*
Consciousness	1/3 (33.33%)	-	2/3 (66.67%)	-	-	-	0.476
Speech disorderDysarthriaAphasia	-3/10 (30%)	1/2 (50%)1/10 (10%)	1/2 (50%)4/10 (40%)	-1/10 (10%)	-1/10 (10%)	--	0.333
Eye findings: Visual field defect Vertical view paralysis	2/2 (100%)-	--	--	--	-1/1(100%)	--	0.247
Sensory deficit	7/45(15.56%)	3/45(6.67%)	22/45(48.89%)	1/45(2.22%)	6/45(13.33%)	6/45(13.33%)	0.264
Motor deficit	6/24 (25%)	1/24 (4.17%)	13/24(54.17%)	1/24(4.17%)	1/24(4.17%)	2/24(8.33%)	0.788
Cerebellar findings	1/3(25%)	-	2/3 (25%)	-	-	-	0.925
Pyramidal findings	7/20 (35%)	-	9/20 (45%)	1/20 (5%)	1/20 (5%)	2/20 (10%)	0.306
Extrapyramidal system uptake findings	-	-	1/2 (50%)	-	1/2 (50%)	-	0.627
Gait disorder	1/6 (16.67%)	-	4/6 (66.67%)	-	1/6 (16.67%)	-	0.643

Comparing the nuclei of the thalamus with the MMSE mean score, we see that the anterior thalamic nuclei differ from other nuclei. In the subgroup values, it was seen that this differentiation was most prominent in the areas of orientation, recall and language (Supplemantery material 1).

When we examined the comparison of the two groups as the classical area and the variative area: the most common cause of the etiological causes of the classic area infarctions was undetermined causes (45.10%) and the second most common cause was major vascular disease (19.61%); while the most common cause of the variative infarction areas was the undetermined causes (31.58%) and major vascular disease (21.05%), cardio embolic stroke (21.05%), small vascular disease (21.05%) were etiologically found in the same ratio. However, there was no statistically significant difference between the two groups (p = 0.297). When the neurological sequela findings of the classical and variative areas were compared, there was no significant difference (p > 0.05). 

When we examined the groups by separating them according to the isolated infarction areas one by one; there were no significant difference in terms of age, disease time and gender in the groups (p = 0.987, p = 0.939, p = 0.076). According to the infarction areas, 20% (n: 14) of the infarctions were anterior, 5.7% (n: 4) are paramedian, 50% (n: 35) are inferolateral, 4.3% (n: 3) are anteromedian, 11.4% (n: 8) were central and 8.6% (n: 6) were posterolateral area infarctions (Table 1). When stroke etiologies according to the vascular areas were evaluated according to the TOAST (Trial of Org 10172 in Acute Stroke Treatment) classification, in inferolateral, the most common infarction area, undetermined etiological cause is the most common 16/35 (45.71%) and the second most common cause in the inferolateral area was large vascular disease being 7/35 (20%); however, this was not statistically significant (p = 0.431) (Table 3).

**Table 3:** Etiological causes of strokes by infarction area.

	Anterior	Paramedian	Inferolateral	Anteromedian	Central	Posterolateral	p*
Major vascular disease	2/14 (14.29%)	2/4 (50%)	7/35 (20%)	2/3 (66.67%)	1/8 (12.5%)	0	0.431
Cardio embolic stroke	2/14 (14.29%)	0	6/35 (17.14%)	0	2/8 (25%)	2/6 (33.33%)	
Small vascular disease	1/14 (7.14%)	0	2/35 (5.71%)	0	3/8 (37.5%)	1/6 (16.67%)	
Other causes	2/14 (14.29%)	1/4 (25%)	4/35 (11.43%)	0	0	1/6 (16.67%)	
Undetermined	7/14 (50%)	1/4 (25%)	16/35 (45.71%)	1/3 (33.33%)	2/8 (25%)	2/6 (33.33%)	

* Chi-square test.

## 4. Discussion

Considering that the cause of the clinical diversity of thalamus infarctions is related to the difference of infarction areas, we aimed to examine the sequela findings of isolated thalamus infarctions one by one according to the classical and variative areas of the vascular lesion areas in this study, and significant association was detected between anterior area infarctions and cognitive impairment. This differentiation in anterior nucleus was most prominent in the areas of orientation, recall and language comparing other areas of thalamus. However, in contrast to our hypothesis, no significant difference was found between sequela clinical findings of other areas. 

As far as we know, the relationship between the anatomical areas of isolated infarctions of the thalamus and their sequela findings has not been studied. In the literature, only acute infarction findings of the topographic areas of the thalamus have been evaluated.In a study with 71 patients, conducted by Carrera et al., 30% of patients were found to have variative area infarction [3]. In our study, similarly, variative area infarction was present in a ratio of one third. Polar artery is a branch of the tuberothalamic artery and its lesion leads to infarction in the anterior of the thalamus, in acute infarctions of this region, orientation disorder, learning disability, memory impairments, personality changes, apathy, abulia develop [5,12,13]. In a study in which twelve patients were prospectively followed up, all patients who had acute anterior thalamic infarction were reported to have cognitive impairments and retrograde amnesia [14]. It is considered that this being associated with anterior nucleus, hippocampus, mammillothalamic tract and prefrontal cortex, and being a part of Papez circuit explains cognitive disorders observed in anterior thalamic infarctions [15–18]. Our study did not evaluate acute findings, however in accordance with the literature, it was detected that those with anterior thalamic area infarction had sequela cognition impairments and MMSE was significantly lower than other area infarctions. This supported that the anterior nucleus is an anatomical structure associated with cognitive functions such as memory, attention, language, and visual spatial functions. 

In the study conducted by Carrera et al., sensory defect was found to be the most common finding in inferolateral area infarctions [3]. In our study, although the most common sequela finding found in the inferolateral area was sensory defect, it was not statistically significant. The reason why it is different from the study conducted by Carrera et al. can be explained by the fact that our findings are sequela and the sensory findings have improved over time. However, our patients presented to hospital with sensory complaints most frequently in the acute infarction period and this finding was also found statistically significant. In the same study, they emphasized that the anteromedian region, which includes anterior and paramedian areas, was associated with cognitive function impairment [3] and they found contradictory results compared to other studies claiming that mammillothalamic tractus and dorsomedial nucleus are affected in cognitive function impairment[12–14,19–21]. In our study, memory impairment was not detected in patients with anteromedian infarction and no significant difference found in MMSE than other field infarctions.

In a study investigating the clinical findings of isolated thalamic infarctions, infarction was the most commonly detected in thalamogeniculate artery area and it was reported that the most common etiological cause was small vascular disease [4]. Also, in our study, the most common infarction area was detected in the inferolateral area fed by thalamogeniculate artery. However, our most common etiological cause was undetermined and the second most common cause was large vascular disease. In this study, it was reported that paramedian infarctions had more neuropsychological, oculomotor, and consciousness disturbances, polar artery infarction area was found along with sensorimotor deficit in frontal-like syndrome and visual field defect was associated with posterior choroidal artery infarction area. Two-thirds of these acute symptoms have been reported to decrease in follow-up [4]. The varying ratios of these sequela findings may explain its difference from our study. Also in this study, unlike our study, bilateral thalamic infarctions were included. 

In a study investigating the variative thalamic area infarcts, 26% of the thalamus infarctions were found to be variative infarcts, and the most common etiological cause was reported to be cardio embolic stroke. They reported that those with unilateral variative infarction most frequently showed cognitive dysfunction. In this study, bilateral multiple variant infarctions of the thalamus and clinical and etiological examinations of variative and classical multiple infarctions were compared. As a result, they reported that thalamus infarctions that presented with various clinical findings were associated with multiple variative topographic maps [11]. In our study, unilateral isolated thalamus infarctions were evaluated, and no significant difference was found between the clinical findings of classical and variative areas. However, since our study contains sequela findings, the improvement of many neurological symptoms may explain this difference. When the etiological causes are examined, in our study, the most common reason among the etiologies of which the causes were detected was found to be large vascular disease. There was also no etiologically significant difference between classical area infarctions and variative area infarctions. 

In a study evaluating the clinical findings of paramedian thalamic infarctions, 12 unilateral and 9 bilateral paramedian infarctions were included in the study. In 5 patients with bilateral paramedian infarction and 4 patients with unilateral paramedian infarction, coma, vertical eye movement impairment and memory impairment were detected. As a result, they reported that paramedian infarctions leads to coma, vertical eye movement impairment and memory impairment in both unilateral and bilateral groups [22]. In another study, it was reported that patients with paramedian infarction had hypersomnia, lack of daytime wakefulness and impaired night sleep stages [23]. In our study, we had 4 patients with paramedian infarction, sensory deficits were found to be the most frequent among the sequela findings of these patients, and no consciousness impairment, memory impairment, eye movements impairment, or hypersomnia were found. Among these patients’ complaints during admission to the hospital, no complaints explaining these findings were reported. This difference can be explained by the small number of patients with paramedian infarction.

In central area infarcts, Carrera et al. reported that all patients had sensory defects and one patient developed vertical view impairment, and that this finding was associated with the median part of the ventral posterolateral nucleus being affected [3]. In our study, sensory deficit was also detected in all patients with central area infarction. Aphasia developed in one patient and parkinsonism findings developed in one patient. However, in our study, the number of patients was also not sufficient to be able to evaluate the clinical findings of central infarcts. Although this situation is explained by the rarity of infarctions in this area, more patients need to be evaluated in order to clarify the information about this area.

Sensory deficit is the most important symptom of patients with posterolateral infarctions because of the presence of ventral posterolateral nucleus in the posterolateral part of the thalamus [24,25]. Because the ventrolateral nucleus is located here, ataxia can be observed in these patients [26]. In our study, sensory deficits were found in all patients with posterolateral area infarction, in accordance with the literature, and vertical view paralysis was detected in one patient. However, ataxia was not detected in this patient group.

Our study has some limitations. Since the number of topographic areas evaluated in the study are high, although the number of patients was sufficient, the number of patients evaluated per area was low in subgroup analyzes. Since this is a study including the last 10 years, the etiologies of the patients reflect the conditions of this period. Therefore, sufficient etiological research may not be performed in each patient according to current conditions. This situation explains the high number of patients with undetermined etiology. 

## 5. Conclusion

In conclusion, patients with anterior area infarction show cognitive sequela findings. Acute period findings of the thalamus infarctions decrease as a result of long-term follow-up. For this reason, in order to be able to evaluate the sequela findings of the future studies, studies in which regular follow-ups are carried out especially in the first few years after the stroke will show the prognosis course of the disease. However, sinceour study does not have an exactly similar one in the literature; its importance is that this study will shed a light on future studies.
